# Glycemic index, glycemic load, dietary inflammatory index, and risk of infertility in women

**DOI:** 10.1002/fsn3.3584

**Published:** 2023-08-09

**Authors:** Behnaz Aghaei, Fardin Moradi, Davood Soleimani, Mehdi Moradinazar, Tina Khosravy, Mehnoosh Samadi

**Affiliations:** ^1^ Student Research Committee, Department of Nutritional Sciences, School of Nutrition Sciences and Food Technology Kermanshah University of Medical Sciences Kermanshah Iran; ^2^ Research Center for Environmental Determinants of Health (RCEDH), School of Public Health Kermanshah University of Medical Sciences Kermanshah Iran; ^3^ Behavioral Disease Research Center Kermanshah University of Medical Sciences Kermanshah Iran; ^4^ Department of Health Nutrition Lorestan University of Medical Sciences Khorramabad Iran

**Keywords:** dietary inflammatory index (DII), glycemic index, glycemic load, infertility, inflammation

## Abstract

The dietary glycemic load (GL) indicates the quantity and quality of carbohydrates, which can affect ovulation and fertility by controlling insulin sensitivity. Also, past studies confirm the role of the dietary inflammatory index (DII) in many diseases, including metabolic syndrome and cardiovascular disorders, so it may be related to reproductive health. This case–control study aims to study the association between glycemic index (GI), GL, and DII with infertility in women. This study was conducted on 300 infertile women in the case group and 300 fertile women in the control group in Kermanshah, Iran. Food intake was evaluated using FFQ, and using NUTRITIONIST IV software programs, GI and GL values were determined. DII was computed as well using FFQ data. Physical activity was assessed using IPAQ‐SF. The association between GI, GL, and DII with infertility was evaluated using a logistic regression test, using STATA version 14 software. The results showed that the DII, GI, and GL were higher in the case group compared to the control group ([*p* = .009], [*p* = .0001], and [*p* = .0007], respectively). The increase in GI, GL, and DII caused an increase in infertility factors, and consequently enhanced chance of infertility ((adjusted odd ratio [OR] 2; 95% confidence interval [CI], 1.16, 3.45), (OR 3.68; 95% CI, 1.99, 6.82), and (OR 1.7; 95% CI, 0.97, 2.95), respectively). The present study indicated that the chance of infertility is higher in women who follow a diet with high GI, GL, and DII. Therefore, a positive association may be present between GI, GL, and DII with infertility.

## Graphical abstract

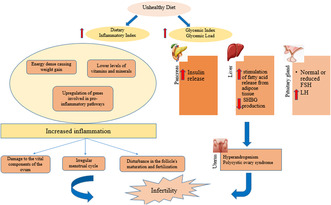

## 1 INTRODUCTION

Infertility is described as the couple's failure to conceive within 1 year for women under 35 years old or 6 months for women over 35, despite unprotected and regular sex (3–4 times a week) (Jafar zadeh‐Kenarsari et al., 2021). As a considerable public health concern, infertility affects women from various emotional, social, and economic aspects, so these people report infertility as one of the most stressful experiences in their lives (Iordăchescu et al., 2021). In recent years, the prevalence of infertility has augmented (Brugo‐Olmedo et al., 2001). A meta‐analysis and systematic review conducted in 2021 shows that infertility affects about 10%–12% of couples worldwide (Kiani et al., 2021). Also, the prevalence of infertility in Iran has been reported as 13.2% (Rakhshanimehr et al., 2020). Every year, infertility disorders increase by an average of 15% (Akhondi et al., 2013). Various factors are related to the pathogenesis of infertility, and several factors often lead to infertility (Zarea et al., 2016). These include polycystic ovary syndrome (PCOS), hormonal disorders, premature ovarian failure, endometriosis or other medical complications (diabetes and thyroid disorders) (Benksim et al., 2018), age, menstrual disease, environmental factors, poor lifestyle choices such as eating habits, smoking, drinking alcohol, and obesity (Tamrakar & Bastakoti, 2019). Nutrition can significantly alter fertility‐related outcomes in men and women (Aoun et al., 2021). However, no solemn guidelines exist for couples of reproductive age (Gaskins & Chavarro, 2018). The studies on the association between carbohydrates and infertility indicate that both quantity and quality of dietary carbohydrates affect glucose metabolism and insulin sensitivity in healthy individuals as well as in diabetics and women with polycystic ovary syndrome (PCOS). Insulin sensitivity may be a primary factor in ovulation and fertility (Chavarro et al., 2009). Some researchers have found that higher HbA1c values are associated with metabolic features similar to PCOS and reduced fertility among apparently healthy women (Hjollund et al., 1999). In spite of the presence of many studies about the effect of insulin sensitivity in regulating female fertility, there are few data on the effect of carbohydrate intake and GL on fertility. Therefore, it seems necessary to conduct more studies in order to obtain more information about the association between the quality and quantity of dietary carbohydrates (i.e., GI, GL) and female infertility.

The dietary inflammatory index (DII) is a new procedure created to estimate the inflammatory capacity of foods. It is associated with inflammatory markers such as interleukin (IL)‐2, IL‐1β, IL‐6, tumor necrosis factor (TNF‐α), and C‐reactive protein (CRP) (Canto‐Osorio et al., 2020). According to scientific evidence, inflammation plays a vital role in the pathology of cancer, cardiovascular disease, and obesity (Pearson et al., 2003; Thun et al., 2004). Studies indicate the essential role of diet in regulating chronic inflammation (Cavicchia et al., 2009; Chrysohoou et al., 2004; Dalziel et al., 2006). Despite numerous studies on the association between DII and various diseases such as metabolic syndrome, cardiovascular diseases, obesity, and type 2 diabetes, the association between DII and female infertility in women has only been investigated in one cohort study yet, and we investigated this association for the first time in a case–control study. Thus, the aim of the study is to discuss the association between GI, GL, and DII with infertility.

## 2 MATERIALS AND METHODS

### 2.1 Study design and data collection

This is a case–control study that was carried out by 600 married women aged 20–40 years in Kermanshah, Iran. In the case group, women referred to the infertility clinic who have problems such as ovulation disorders, irregular menstruation, menstrual bleeding disorders, PCOS, hirsutism, overweight, and obesity (body mass index [BMI] more than 25 kg/m^2^) after being approved by a gynecologist. They were included in the study. These women had at least one ovary and one healthy fallopian tube and had not significantly changed their diet during the last 6 months. Exclusion criteria include the following: history of underlying diseases such as thyroid disorders, diabetes, high blood pressure, high blood lipids, cancer, secondary infertility, infertility with unknown cause, male infertility (the cause of infertility is not related to women), presence of tubal factor, endometriosis, use of contraceptives, multivitamin and mineral supplements, alcohol, and tobacco. The duration of infertility was at least 1 year in women younger than 35 years and at least 6 months in women older than 35 years. The cause of infertility was diagnosed after examining the patient's medical record and confirming it by a gynecologist. After being diagnosed with infertility, 300 women were included in the study as cases. The sampling technique was conducted according to Ness et al. (2000). Three hundred people were included in each of the case group and control group. The sample size has 0.8 power, with a significance of 0.05 type I error.

The samples of the control group were collected from available women in the city. These women experienced at least one history of successful pregnancy who have finished breastfeeding and whose fertility was not artificial. In this stage, we tried to match case and control groups based on anthropometric variables, physical activity level, age, economic status, and education level, then for each case, a control without infertility was included in the study. Data collection took 11 months. First, the infertile women data were collected during 7 months, from June 2021 to January 2022, and then, the fertile women data were collected during 4 months, from January to May 2022. The participants completed demographic questionnaire (age, educational level, occupation, family income, smoking, etc.), dietary intake (semiquantitative food frequency questionnaire), and physical activity questionnaire (International Physical Activity Questionnaire [IPAQ]).

### 2.2 Measurements

We determined the height with a tape measure with a precision of 0.1 cm and weight with minimum clothes and without additional items by Seca digital scale with an accuracy of 100 g. The body mass index (BMI) was determined as body weight in kilograms/height in square meters (normal range: 18.5–24.9 kg/m^2^) (World Health Organization, 2004). Also, the waist circumference (WC) was measured using a none elastic tape measure in the smallest area between the last rib and the iliac crest (Normal range: 80–88 cm) and hip circumference (HC) using a none‐ flexible tape measure in the most significant space between the waist and knees. Then, the waist‐to‐hip (WHR) and WHtR (WC/height) were determined (normal value: ≤0.85 & 0.40–0.50, respectively) (Hertelyova et al., 2016).

### 2.3 Food intake assessment

Food intake assessment was done by using a 167‐item semiquantitative food frequency questionnaire (FFQ), which indicates diet during the last year. This questionnaire evaluated the consumption of food items such as milk and dairy products, legumes, fruits, vegetables, meat and its derivatives, bread and cereals, different types of oil and oilseeds, sugar and seasonings, snacks, sweets and desserts, nuts, mayonnaise, dried fruit, buttermilk, soda, and salt (Esmaillzadeh et al., 2007; Mirmiran et al., 2009). Previous studies have confirmed the validity and reliability of this questionnaire (Keshteli et al., 2014). After introducing each food group's units and consumption units to the participants, the researcher completed this questionnaire face to face. By using NUTRITIONIST IV (N4) software program, energy, macronutrient, and micronutrient values were determined. GI ranks carbohydrates based on how they affect blood sugar relatively. Simple carbohydrates elevate blood glucose, so they have a higher GI, while high‐fiber foods reduce blood glucose, so they have a lower GI (Zirak Sharkesh et al., 2022). To determine the average daily GI of each person, the GI of one serving of each foodstuff was multiplied by the average number of servings of that foodstuff and the results were added together; then, this value was divided by the total value of available carbohydrates (Noli et al., 2020).

$$\begin{array}{c} \mathrm{GI}=\text{Total}\;\left(\mathrm{GI}\;\text{of each food item}\times \text{available carbohydrates of that food item}\right)/\\ \;\text{total available carbohydrates} \end{array}$$

The USDA Food Composition Table acquired the total carbohydrate and fiber. Due to missing data, Iran's GI table can extract only six GI values from 85 carbohydrate‐containing foods (Taleban & Esmaeili, 1999). While GI values for 62 other foods were selected from international tables (Atkinson et al., 2008) and the GI values for the remaining 17 foods were estimated based on similar foods in physical and chemical terms. The dietary GL was determined using the following formula and expressed as g/d (Haghighatdoost et al., 2016; Sajjadi et al., 2019).

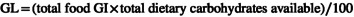




The DII is a scoring algorithm developed by Shivapa et al. examining the inflammatory potential of 45 foodstuffs (Shivappa et al., 2014). +1 score for dietary parameters that increase IL‐6, TNF‐α, IL‐1β, CRP and decrease IL‐4, IL‐10, −1 Score for dietary parameters that increase IL‐4, IL‐10 and decrease IL‐6, TNF‐α, IL‐1β, CRP, and a zero score is considered for food parameters that have no effect on the change of inflammatory factors (Shivappa et al., 2014). Thus, a higher DII score demonstrates more inflammatory potential in the diet (Diba‐Bagtash et al., 2021). Our study determined DII using FFQ, which included 31 of the 45 possible nutrients in the main DII. Nine of 31 parameters were pro‐inflammatory and increased the DII score, including iron, vitamin B‐12, total energy intake, protein, carbohydrate, fat, cholesterol, and saturated and trans fatty acids. Twenty‐two parameters had an anti‐inflammatory effect. They reduced the DII score, including alcohol, vitamin B‐6, beta‐carotene, caffeine, fiber, folic acid, garlic, magnesium, Monounsaturated Fatty Acids, niacin, Polyunsaturated Fatty Acids, n‐6 fatty acids, riboflavin, selenium, thiamin, zinc, vitamin A, n‐3 fatty acids, vitamin D, vitamin C, green/black tea, and vitamin E. To calculate the DII, the intake of each food was determined through the FFQ. Then, the standardized information was determined using the global database and converted into a percentile score (*Z*‐Score). This is obtained by subtracting the “standard average” from the value reported and dividing this value by its standard deviation. Each percentile score is doubled, and then “1” is subtracted. Until a symmetric distribution with 0 centralities is obtained. The central percentile value for each food parameter was then multiplied by the corresponding “Total Food Parameter Specific Inflammatory Effect Score” to acquire the “Food Parameter Specific DII Score.” Finally, these scores are summed to create an individual's “total DII score” (Diba‐Bagtash et al., 2021). DII = b1*n1 + b2*n2……b31*n31, where b refers to the literature‐derived inflammatory effects score for each of the assessable food parameters, and n refers to the food parameter‐specific centered percentiles, which were obtained from this case–control's dietary data.

### 2.4 Physical activity assessment

The short‐form IPAQ questionnaire assessed the participant's physical activity. Previous studies have confirmed the validity and reliability of this questionnaire (Moghaddam et al., 2012). It includes a scale ranging from 1 (going from one place to another), 2 (shopping), 3 (working at home), 4 (actions performed in their free time), 5 (sports training). Also, intense physical activities were asked. Answers are recorded in minutes per week. Using IPAQ, a numerical measure for each physical activity, the MET index is the equivalent metabolic index of each physical activity. MET = 1 index means consumption of 1 kilocalorie of energy per kilogram of body weight in 1 h. In the questionnaire analysis, activities were performed with low physical effort and did not cause enhancement in breathing or heart rate as “light activities,” they were performed with moderate physical effort and caused a small increase in breathing or heart rate as “moderate activities” and activities that were performed with high physical effort and caused a large increase in breathing or heart rate were considered as “Severe activities.” For each activity, a separate score was computed, added together, and given zero if accomplished no physical activity. Each person's final physical activity score was For each activity, a separate score was determined, added together, and given zero if accomplished no physical activity. Each person's final physical activity score was calculated using the following formula based on MET‐minutes/week using the following formula based on MET‐minutes/week (IPAQ Research Committee, 2005).

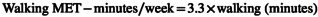

$$\begin{array}{c} \text{Moderate}\;\mathrm{MET}‐\text{minutes}/\text{week}=4.0\times \text{moderate}\\ \;-\text{intensity activity}\;\left(\text{minutes}\right) \end{array}$$

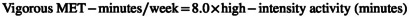




### 2.5 Data analysis

Quantitative variables (age, height, weight, BMI, waist circumference, hip circumference, waist to height ratio, waist to hip ratio) were described by mean ± standard deviation (SD), and qualitative variables (level of education, family income, physical activity level) were reported using frequency (%). The participants' baseline characteristics were compared using a *t*‐test for quantitative data and a chi‐square (χ^2^) test for qualitative data. Chi‐square was used to compare the various infertility factors with each of the GI, GL, and DII. The comparison of GI, GL, and DII in the two studied groups was made by *t*‐test. One‐way ANOVA test was used to compare the anthropometric variables, food consumption, and physical activity based on GI, GL, and DII. A logistic regression test assessed the adjusted and multivariate modeling of the association among DII, GL, and GI and infertility risk. All statistical analysis was carried out by STATA version 14 software.

## 3 RESULTS

After data collection, the results were summarized in tables showing the potential associations between the three parameters studied (GI, GL, and DII) and infertility rates in women, alongside the different characteristics of the population.

### 3.1 Demographic and anthropometric characteristics

Table 1 indicates the demographic and anthropometric characteristics and physical activity level of cases and controls. Physical activity, age, educational level, and family income had no statistically significant differences. Physical activity is divided into three categories as follows: low (<600 Met‐minutes/week), moderate (600–3000 Met‐minutes/week), severe (>3000 Met‐minutes/week) (Tran et al., 2013), and moderate physical activity was the most seen among both cases and controls. At the same time, the mean WC, HC, and BMI in the control group were lower than in the case group (*p* < .001).

**TABLE 1:** Comparison of anthropometric characteristics and physical activity level in two groups.

Variable	Infertile women, *N* = 300	Fertile women, *N* = 300	*p*‐Value
	Number (percentage)		
Level of education			
Illiterate	9 (3)	1 (0.33)	.07
Elementary and cycle	110 (36.67)	106 (35.33)	
Diploma	102 (34)	109 (36.33)	
Higher than diploma	79 (26.33)	84 (28)	
Family income (Toman)			
Less than a million	56 (18.67)	37 (12.33)	.13
One to two million	61 (20.33)	58 (19.33)	
Two or three million	62 (20.67)	61 (20.33)	
Three to four million	53 (17.67)	72 (24)	
Above four million	68 (22.67)	72 (24)	
Physical activity level (Met‐minutes/week)			
Low	32 (60.38)	21 (39.62)	.24
Moderate	258 (48.77)	271 (51.23)	
Severe	10 (55.56)	8 (44.44)	

Mean ± SD			
Age (year)	32.46 ± 5.6	32.57 ± 5.1	.80
Height (cm)	161 ± 0.05	162 ± 0.05	**.001**
Weight (kg)	13.7 ± 73.42	70.47 ± 11.8	**.005**
BMI (kg/m^2^)	28.21 ± 4.6	26.61 ± 4.2	**<.001**
Waist circumference (cm)	90.77 ± 8.9	87.37 ± 10.8	**<.001**
Hip circumference (cm)	111.96 ± 8.8	107.82 ± 9.6	**<.001**
WHtR (waist to height ratio)	0.55 ± 0.05	0.53 ± 0.06	.39
WHR (waist to hip ratio)	0.80 ± 0.04	0.80 ± 0.06	.92

*Note*: *p*‐Values were acquired for quantitative data using *t*‐test and for qualitative data using chi‐square (χ^2^). *p*‐Value <.05 is considered statistically significant (in bold).

### 3.2 GI, GL, and DII in the two groups studied

Based on the comparison of GI, GL, and DII in fertile and infertile women, GI, GL, and DII in infertile women were more than the fertile women (*p* = .0001, .0007, and .009, respectively) (Table 2).

**TABLE 2:** Comparison of glycemic index, glycemic load, and dietary inflammation index in two studied groups.

Variable	Infertile women, *N* = 300	Fertile women, *N* = 300	*p*‐Value
	Mean ± SD		
GI	51.49 ± 5.6	49.78 ± 4.9	**.0001**
GL	132.43 ± 45.8	118.78 ± 52.5	**.0007**
DII	−0.82 ± 1.6	−1.15 ± 1.4	**.009**

*Note*: The data are expressed as the mean ± standard deviation and the *p*‐values were acquired from the *t*‐test. *p*‐Value <.05 is considered statistically significant (in bold).

### 3.3 Investigation of anthropometric variables and food intake based on GI and GL changes

After comparing food intake and anthropometric variables based on GI and GL, GI showed a significant association with carbohydrate consumption in cases and controls (higher among cases) (*p* < .001 and *p* = .02, respectively), when carbohydrate intake increased, the GI increased as well. As for the GL, a significant association was found between GL and energy and all macronutrients (*p* < .001). No significant association was found among the anthropometric variables with GI and GL (Table 3).

**TABLE 3:** Comparison of anthropometric variables and food intake based on dietary glycemic index and glycemic load in two studied groups.

Variable	GI (Mean ± SD)				*p*‐Value	GL (Mean ± SD)				*p*‐Value
	1 (lowest quarter)	2	3	4 (highest quarter)		1 (lowest quarter)	2	3	4 (highest quarter)	
Energy (kcal/day)										
Infertile	2343.10 ± 741.4	2539.26 ± 775.4	2666.58 ± 715.6	2577.42 ± 850.4	.05	1637.73 ± 447.1	2054.22 ± 364.3	2573.03 ± 444.1	3272.61 ± 533.7	**<.001**
Fertile	2255.68 ± 86.89	2180.51 ± 576.8	2187.88 ± 681.9	2372.13 ± 89.53	.33	1565.77 ± 462.3	2141.30 ± 40.56	2572.83 ± 500.6	3254.95 ± 691.3	**<.001**
Protein (g/day)										
Infertile	90.66 ± 38.2	91.44 ± 33.8	92.66 ± 31.3	83.62 ± 31.0	.45	59.60 ± 23.1	72.14 ± 19.6	93.35 ± 24.8	116.27 ± 32.4	**<.001**
Fertile	84.79 ± 37.8	80.44 ± 25.4	75.68 ± 27.3	81.43 ± 35.7	.40	56.83 ± 21.0	78.97 ± 25.5	89.10 ± 23.4	112.4 ± 34.7	**<.001**
Carbohydrate (g/day)										
Infertile	302.10 ± 92.9	335.37 ± 96.3	368.47 ± 101.2	376.69 ± 124.9	**<.001**	206.83 ± 46.2	273.50 ± 27.5	345.55 ± 39.0	457.46 ± 64.6	**<.001**
Fertile	285.47 ± 99.1	278.88 ± 82.5	290.16 ± 96.0	325.63 ± 135.7	**.02**	196.18 ± 42.4	273.45 ± 38.5	338.66 ± 40.2	464.54 ± 110.8	**<.001**
Fat (g/day)										
Infertile	89.96 ± 35.4	97.13 ± 37.4	95.61 ± 32.6	85.36 ± 34.5	.19	66.68 ± 29.1	78.70 ± 27.3	95.16 ± 32.2	113.4 ± 32.0	**<.001**
Fertile	93.18 ± 49.4	87.30 ± 31.7	85.03 ± 31.9	86.0 ± 34.0	.60	65.43 ± 32.8	85.97 ± 27.6	110.77 ± 30.6	110.87 ± 39.3	**<.001**
BMI (kg/m^2^)										
Infertile	27.65 ± 3.7	28.92 ± 4.7	27.55 ± 4.4	28.58 ± 5.1	.19	27.62 ± 4.3	28.53 ± 4.5	28.30 ± 5.0	28.56 ± 4.6	.52
Fertile	26.45 ± 4.1	26.53 ± 4.8	26.27 ± 4.0	27.40 ± 3.8	.45	26.23 ± 3.9	25.82 ± 3.9	27.46 ± 4.6	26.64 ± 4.2	.12
WC (cm)										
Infertile	89.85 ± 7.8	92.22 ± 9.8	89.85 ± 8.6	91.01 ± 9.1	.33	89.59 ± 9.0	91.74 ± 9.4	90.14 ± 7.2	92.01 ± 9.8	.26
Fertile	86.58 ± 10.3	86.21 ± 12.5	88.23 ± 10.4	89.20 ± 9.8	.32	87.15 ± 9.5	84.95 ± 9.7	89.09 ± 12.4	87.58 ± 10.8	.16
WHtR										
Infertile	0.55 ± 0.05	0.56 ± 0.05	0.54 ± 0.05	0.56 ± 0.05	.16	0.55 ± 0.06	0.56 ± 0.05	0.55 ± 0.04	0.56 ± 0.06	.64
Fertile	0.52 ± 0.06	0.52 ± 0.07	0.53 ± 0.06	0.54 ± 0.06	.22	0.52 ± 0.05	0.51 ± 0.06	0.54 ± 0.08	0.53 ± 0.06	.22
WHR										
Infertile	0.80 ± 0.04	0.80 ± 0.04	0.80 ± 0.04	0.80 ± 0.04	.54	0.80 ± 0.04	0.80 ± 0.04	0.79 ± 0.04	0.81 ± 0.04	.41
Fertile	0.80 ± 0.06	0.79 ± 0.07	0.82 ± 0.06	0.80 ± 0.07	.08	0.80 ± 0.06	0.80 ± 0.07	0.80 ± 0.07	0.80 ± 0.06	.90

*Note*: Data are expressed as mean ± standard deviation and *p*‐values were acquired using one‐way ANOVA test. *p*‐Value <.05 is considered statistically significant (in bold).

### 3.4 Investigation of anthropometric variables and food intake based on DII changes

After comparison of DII on different quartiles, against the different variables, DII increased significantly with an increase in energy, carbohydrates, and fats, while it decreased with high protein intakes (*p* < .001). Also, higher BMI, WC, and WHtR were associated with a significant increase in DII in the control group (*p* < .001, *p* = .004, and *p* = .001, respectively) (Table 4).

**TABLE 4:** Comparison of anthropometric variables and food intake based on dietary inflammatory index in two studied groups.

Variable	DII (Mean ± SD)				*p*‐Value
	1 (lowest quarter)	2	3	4 (highest quarter)	
Energy (kcal/day)					
Infertile	1614.48 ± 416.3	2292.57 ± 464.0	2753.83 ± 596.0	3120.76 ± 685.4	**<.001**
Fertile	1638.07 ± 511.3	2207.20 ± 529.8	2389.56 ± 451.19	2961.63 ± 818.6	**<.001**
Protein (g/day)					
Infertile	115.72 ± 32.4	101.97 ± 27.9	77.68 ± 19.9	52.39 ± 14.1	**<.001**
Fertile	107.09 ± 32.3	87.39 ± 21.3	79.52 ± 26.1	54.59 ± 19.4	**<.001**
Carbohydrate (g/day)					
Infertile	218.76 ± 62.5	316.66 ± 64.0	373.56 ± 87.1	416.19 ± 96.8	**<.001**
Fertile	214.01 ± 59.3	280.53 ± 66.5	323.26 ± 78.2	398.31 ± 123.9	**<.001**
Fat (g/day)					
Infertile	60.59 ± 20.1	82.68 ± 25.5	99.34 ± 31.5	116.81 ± 35.2	**<.001**
Fertile	65.07 ± 30.1	89.02 ± 23.6	89.28 ± 32.1	111.54 ± 40.8	**<.001**
BMI (kg/m^2^)					
Infertile	27.52 ± 4.7	27.91 ± 4.1	28.83 ± 4.8	28.62 ± 4.6	.24
Fertile	25.21 ± 3.4	25.73 ± 3.9	26.39 ± 4.4	28.42 ± 4.2	**<.001**
Waist circumference (cm)					
Infertile	89.43 ± 7.4	89.77 ± 10.0	91.17 ± 7.8	92.61 ± 9.5	.12
Fertile	84.79 ± 10.3	86.30 ± 11.3	86.45 ± 10.5	90.68 ± 10.5	**.004**
Waist to height ratio					
Infertile	0.55 ± 0.06	0.54 ± 0.05	0.56 ± 0.04	0.56 ± 0.06	.20
Fertile	0.51 ± 0.06	0.55 ± 0.06	0.52 ± 0.06	0.52 ± 0.06	**.001**
Waist to hip ratio					
Infertile	0.79 ± 0.04	0.80 ± 0.04	0.80 ± 0.04	0.81 ± 0.04	.45
Fertile	0.79 ± 0.06	0.81 ± 0.07	0.81 ± 0.06	0.79 ± 0.07	.08

*Note*: Data are expressed as mean ± standard deviation and *p*‐values were acquired using one‐way ANOVA test. *p*‐Value <.05 is considered statistically significant (in bold).

### 3.5 Investigating the percentage of different infertility factors based on GI, GL, and DII

Table 5 indicates different infertility factors with GI, GL, and DII, respectively. PCOS, ovulation disorder, irregular menstruation, and menstrual bleeding were higher with an increased GI (*p* = .02, *p* = .001, *p* = .001, *p* = .002, respectively). The same factors were observed with a high GL, in addition to hirsutism (*p* = .01, *p* < .001, *p* < .001, *p* < .001, *p* = .04, respectively). As for DII, the percentage of PCOS and ovulation disorders has increased significantly (*p* = .01 and *p* = .02, respectively).

**TABLE 5:** Comparison of various infertility factors based on changes in glycemic index, glycemic load, and dietary inflammatory index in infertile women.

Infertility factors	GI				*p*‐Value	GL				*p*‐Value	DII				*p*‐Value
	1 (lowest quarter)	2	3	4 (highest quarter)		1 (lowest quarter)	2	3	4 (highest quarter)		1 (lowest quarter)	2	3	4 (highest quarter)	
	Number (percentage)					Number (percentage)					Number (percentage)				
PCOS	35 (23.33)	50 (33.33)	52 (34.67)	60 (40)	.02	21 (23.86)	22 (22.92)	68 (37.16)	86 (36.91)	.01	46 (23.35)	37 (18.78)	51 (25.89)	63 (31.98)	.01
Ovulation disorder	47 (31.33)	63 (42)	73 (48.67)	80 (53.33)	**.001**	23 (26.14)	30 (31.25)	89 (48.63)	121 (51.93)	**<.001**	63 (23.95)	52 (19.77)	71 (27)	77 (29.28)	**.02**
Irregular Menstruation	44 (29.33)	62 (41.33)	69 (46)	77 (51.33)	**.001**	23 (26.14)	27 (28.13)	85 (46.45)	117 (50.21)	**<.001**	61 (24.21)	51 (20.24)	67 (26.59)	73 (28.97)	.06
Menstrual bleeding disorder	42 (28)	49 (32.67)	62 (41.33)	71 (47.33)	**.002**	19 (21.59)	26 (27.08)	82 (44.81)	97 (41.63)	**<.001**	55 (24.55)	46 (20.54)	63 (28.13)	60 (26.79)	.21
Hirsutism	36 (24)	36 (24)	44 (29.33)	51 (34)	.15	19 (21.59)	18 (18.75)	56 (30.60)	74 (31.76)	**.04**	46 (27.54)	32 (19.16)	43 (25.75)	46 (27.54)	.23
Obesity	33 (22)	40 (26.67)	30 (20)	41 (27.33)	.37	18 (20.45)	23 (23.96)	48 (26.23)	55 (23.61)	.77	31 (21.53)	33 (22.92)	48 (33.33)	32 (22.22)	.06
A set of symptoms	2 (1.33)	2 (1.33)	6 (4)	4 (2.67)	.35	1 (1.14)	3 (3.13)	5 (2.73)	5 (2.15)	.80	3 (1.99)	5 (3.36)	2 (1.32)	4 (2.68)	.67

*Note*: The data are expressed as a number (percentage). *p*‐values were acquired using chi‐square (χ^2^). *p*‐Value < .05 is considered statistically significant (in bold).

### 3.6 Investigating the association between GI, GL, and DII with female infertility

Table 6 indicates the association between GI, GI, and DII with the chance of female infertility. GI, GL, and DII were adjusted in three separate models and adjusted with demographic and anthropometric variables, respectively. The odds ratio (OR) and confidence interval for each model were reported and acquired using logistic regression. The presented results acknowledged the possible association between GI, GL, DII, and female infertility. Thus, the OR value in the ** model shows that the chance of female infertility increases by increasing DII (adjusted OR 1.7; 95% CI, 0.97, 2.95), GI (OR 2; 95% CI, 1.16, 3.45), and GL (OR 3.68; 95% CI, 1.99, 6.82). DII was significant in the crude model, fourth quartile (*p* = .03), while GI and GL were significant in the third and fourth quartiles of all three models (*p* = .04, *p* = .01, respectively, for GI in ** model) and (*p* < .001 for GL in ** model).

**TABLE 6:** Multivariate modeling of the association between glycemic index, glycemic load, and dietary inflammatory index and infertility risk.

Variable	Crude model, OR (95% CI)	*p*	Model^a^, OR (95% CI)	*p*	Model^b^, OR (95% CI)	*p*
DII						
First quarter	1	—	1	—	1	—
Second quarter	0.68 (0.43–1.07)	.10	0.73 (0.44–1.22)	.23	0.60 (0.35–1.03)	.06
Third quarter	1.17 (0.74–1.84)	.49	1.19 (0.72–1.98)	.48	1.19 (0.69–2.04)	.52
Fourth quarter	1.62 (1.03–2.57)	**.03**	1.44 (0.86–2.41)	.15	1.7 (0.97–2.95)	.06
GI						
First quarter	1	—	1	—	1	—
Second quarter	1.50 (0.95–2.39)	.08	1.37 (0.82–2.29)	.21	1.37 (0.80–2.36)	.24
Third quarter	1.97 (1.24–3.12)	**.004**	1.64 (0.98–2.75)	**.05**	1.75 (1.02–3.0)	**.04**
Fourth quarter	2.66 (1.67–4.24)	**<.001**	2.08 (1.23–3.52)	**.006**	2 (1.16–3.45)	**.01**
GL						
First quarter	1	—	1	—	1	—
Second quarter	1.16 (0.63–2.14)	.61	1.50 (0.75–3.0)	.24	1.55 (0.76–3.15)	.22
Third quarter	2.56 (1.50–4.35)	**.001**	3.68 (1.97–6.85)	**<.001**	3.45 (1.83–6.52)	**<.001**
Fourth quarter	2.75 (1.64–4.60)	**<.001**	3.56 (1.94–6.51)	**<.001**	3.68 (1.99–6.82)	**<.001**

^a^ Adjusted with demographic variables including age, education level, occupation, family income, and physical activity level.

^b^ In addition to demographic variables, anthropometric indices including BMI, waist circumference, waist‐to‐height ratio, and waist‐to‐hip ratio have also been adjusted. *p*‐Values were acquired using logistic regression test. *p*‐Value <.05 is considered statistically significant (in bold).

Eventually, the results show that the DII, GI, and GL were higher among cases and increased the risk for infertility factors among women.

## 4 DISCUSSION

In this case–control study, we investigated the association between GI, GL, DII, and infertility in women in Kermanshah City. According to our knowledge, this is the first case–control study to investigate the association between DII and female infertility. Also, there are few studies on the association between GI and GL with infertility in women. Preconception nutrition is essential, and evidence suggests that preconception nutritional behaviors may ameliorate embryo and oocyte quality, implantation, and successful maintenance of pregnancy to term (Garruti et al., 2019; Moran et al., 2016).

The results of this study demonstrated that the infertile group has a higher food GI and GL than the control group. Also, with increasing GI and GL, the odds of infertility in women increased. The GI quantifies glycemic responses induced by carbohydrates in various foods (Ludwig, 2002). The GL represents the increase in blood glucose based on the number of carbohydrates in a meal and was introduced to obtain an overall estimate of postprandial glycemia and insulin requirements (Salmerón et al., 1997; Zeinali et al., 2016). A prospective cohort study indicating that diets with high GL, carbohydrate‐to‐fiber ratio, and added sugar are associated with reduced fertility (Willis et al., 2020). A randomized controlled study showed that a low‐calorie diet with low GI and GL reduces weight, BMI, body fat percentage, and leptin concentration, which bettered the pregnancy rate (Becker et al., 2015). The prospective study by Chavarro et al. (2009) concluded that the quality and quantity of carbohydrates in the diet are the main characteristics of ovulation and fertility in females, and infertility due to lack of ovulation has a positive association with total carbohydrate intake and GI. The results of another study indicate that a high HbA1C was directly associated with testosterone and indirectly with inhibin A. As a result, the reduction in fertility among women with high HbA1C may be related to subclinical polycystic ovaries, as indicated by the hormonal profile (Hjollund et al., 1999).

The high concentration of insulin can regulate the production of free testosterone and lead to hyperandrogenism through the increase of free insulin‐like growth factor I (IGF‐I) and sex hormone‐binding globulin (SHBG). Thus, it may alter the maturation of oocytes (Silvestris et al., 2019), This can justify insulin resistance's role as a determining factor in ovulation function (Fica et al., 2008). According to the results, both the amount and type of carbohydrates are undoubtedly significant in the context of a fertility‐supporting diet, especially among women who intend to become pregnant and in whom there is evidence of carbohydrate and lipid metabolism disorders.

According to our findings, participants in the case group had higher DII than the control group. Also, the odds of infertility and the percentage of diseases related to infertility increased with increasing DII, although it was not statistically significant for the odds of infertility. A recent review suggested that dietary interventions that reduce inflammation in men and women during preconception may improve fertility outcomes. This review also highlights the critical gaps in the role of diet in improving fertility and outlines recommendations for future study in this field (Alesi et al., 2022). In a recent retrospective cohort study, it was shown that the odds ratio of infertility in the fourth quartile was 1.48 times higher than in the first quartile of DII (Moludi et al., 2023). Another study in India examined the degree of primary infertility and its contributing factors in 402,807 married women. According to the results of this study, infertility in women was related to diet and the positive effect of dark green leafy vegetables, fruits, and milk/curd was reported (Unisa et al., 2022). A case–control study in Tehran observed that the probability of abortion was higher in subjects who consumed a diet with a higher DII than those with a lower pro‐inflammatory potential (Vahid et al., 2017). In the study of Jahangirifar et al. (2019), the relationship between diet and fertility outcomes in infertile women was investigated. The findings of this study showed that following a healthy diet may increase oocyte quality and quantity. Also, an unhealthy diet can negatively affect the chances of fertility. In two case–control studies, the risk of PCOS increased with increasing DII (Wang et al., 2022; Zirak Sharkesh et al., 2022). PCOS is one of the diseases associated with infertility and inflammation, which leads to an increase in inflammatory markers such as CRP, IL‐18, the number of white blood cells, monocyte chemoattractant protein‐1 (MCP‐1), and macrophage inflammatory protein α‐1 (MIP‐1α), as well as changes in the expression of IL‐6 and TNF‐α (Rudnicka et al., 2021). Despite this, in a cross‐sectional study on 144 infertile women (Diba‐Bagtash et al., 2021), it was shown that DII was not associated with any parameter of treatment outcome related to IVF treatment in infertile women. A systematic review also concluded that there is insufficient evidence to support the recommendation of a dietary pattern to improve pregnancy or live birth rates in women undergoing IVF (Sanderman et al., 2022).

The increase of inflammatory markers is responsible for many harmful effects of DII (Canto‐Osorio et al., 2020). The association with an anti‐inflammatory diet, which contains rich resources of β‐Carotene, vitamin C, omega‐3, and fiber, has been shown to reduce inflammatory factors such as CRP and IL‐6, respectively (Ahluwalia et al., 2013). According to scientific evidence, chronic inflammation is an essential mechanism that hurts women's fertility through contributing to irregular menstrual cycles, damage to the vital components of the ovum, hormonal cycle, disturbance in the process of maturation and fertilization of follicles, as well as disturbance in embryo implantation (Ayeneh pour et al., 2020; Vaisi‐Raygani & Asgari, 2021). The inflammation prevents decidualization, decreases progesterone levels, and disrupts endometrial function also increases aromatase activity and makes an estrogen‐dominant phase (Rasheed & Hamid, 2020). Reactive oxygen species also have negative effects on the endometrium, oocyte, and sperm and affect the molecular level by causing oxidative stress. The antioxidants in fruits and vegetables can significantly increase the antioxidant capacity of plasma, and in this way, it can positively affect the percentage of oocytes and the quality of second‐stage oocytes (Harasym & Oledzki, 2014). Furthermore, blood coagulation plays an essential role in the interaction of the embryo with the endometrium, and a coagulation defect disrupts the implantation of the source. According to scientific evidence, inflammation can affect fertility through its effect on blood coagulation because inflammatory cytokines can increase blood coagulation and thrombosis (Joseph et al., 2002). Coagulation changes are often reported in pro‐inflammatory conditions such as endometriosis, PCOS, and adenomyosis (Gerotziafas et al., 2017). We found that diet can affect fertility potential through different pathways, but there is still extensive debate among researchers about the cause of infertility, which is multifactorial and there is a need for more research in this field.

The present study has limitations. As mentioned, in this study, the FFQ questionnaire was used to evaluate the subject's food intake, which depends on memory. Food intake may be reported as less or more than reality. Also, it did not investigate the association between GI, GL, and DII and hormonal and metabolic characteristics. In addition, the classification of PCOS patients into different phenotypes was not done based on the Rotterdam criteria, and future studies are suggested to consider different PCOS phenotypes. Also, due to its case–control design, it was not possible to examine the causal relationship between DII and the progress of infertility.

## 5 CONCLUSION

The present case–control study showed that adherence to a diet with high GI, GL, and DII increases the risk of infertility in women. This association can be explained by the disruption in glucose and insulin metabolism and increased inflammation, which have adverse effects on women's reproductive health. Reducing the consumption of simple carbohydrates and saturated and trans fatty acids is associated with reducing the risk of chronic diseases. Therefore, to prevent and reduce the risk of infertility, women can be advised to reduce the consumption of foods with high GI, GL, and DII. However, more studies are needed to confirm these findings.

## AUTHOR CONTRIBUTIONS

**Mehnoosh Samadi:** Conceptualization (lead); data curation (lead); methodology (lead); project administration (lead); supervision (lead); validation (lead); writing – review and editing (lead). **Behnaz Aghaei:** Conceptualization (lead); data curation (lead); formal analysis (lead); investigation (lead); project administration (lead); resources (lead); software (lead); validation (lead); writing – original draft (lead); writing – review and editing (lead). **Davood Soleimani:** Software (equal); validation (equal); writing – review and editing (equal). **Fardin Moradi:** Writing – original draft (equal); writing – review and editing (equal). **Mehdi Moradinazar:** Formal analysis (equal); software (equal). **Tina Khosravy:** Investigation (equal).

## FUNDING INFORMATION

This study was approved and supported by a grant from Kermanshah University of Medical Sciences, Kermanshah, Iran (grant number 4000269).

## CONFLICT OF INTEREST STATEMENT

The authors declare no conflict of interest.

## ETHICS STATEMENT

We registered the current study with tracking number 4000269 in Deputy of Research and Technology, which was approved by the Ethics Committee of Kermanshah University of Medical Sciences (ethical code: IR.KUMS.REC.1400.185). The volunteers entered the study after being informed about the research objectives and providing informed consent. In case of unwillingness to continue cooperation, they could be excluded from the study without a specific reason.

## CONSENT FOR PUBLICATION

All authors have permission to publish this article.

## Data Availability

The datasets used and/or analyzed during this study are available from the corresponding author on reasonable request and permission for use was received by the Ethics Committee of Kermanshah University of Medical Sciences.
